# Synthetic or Food-Derived Vitamin C—Are They Equally Bioavailable?

**DOI:** 10.3390/nu5114284

**Published:** 2013-10-28

**Authors:** Anitra C. Carr, Margreet C. M. Vissers

**Affiliations:** Centre for Free Radical Research, Department of Pathology and Biomedical Science, University of Otago, Christchurch, P.O. Box 4345, Christchurch 8140, New Zealand; E-Mail: margreet.vissers@otago.ac.nz

**Keywords:** ascorbate, dietary vitamin C, bioavailability, animal studies, human studies, bioflavonoids

## Abstract

Vitamin C (ascorbate) is an essential water-soluble micronutrient in humans and is obtained through the diet, primarily from fruits and vegetables. *In vivo*, vitamin C acts as a cofactor for numerous biosynthetic enzymes required for the synthesis of amino acid-derived macromolecules, neurotransmitters, and neuropeptide hormones, and is also a cofactor for various hydroxylases involved in the regulation of gene transcription and epigenetics. Vitamin C was first chemically synthesized in the early 1930s and since then researchers have been investigating the comparative bioavailability of synthetic *versus* natural, food-derived vitamin C. Although synthetic and food-derived vitamin C is chemically identical, fruit and vegetables are rich in numerous nutrients and phytochemicals which may influence its bioavailability. The physiological interactions of vitamin C with various bioflavonoids have been the most intensively studied to date. Here, we review animal and human studies, comprising both pharmacokinetic and steady-state designs, which have been carried out to investigate the comparative bioavailability of synthetic and food-derived vitamin C, or vitamin C in the presence of isolated bioflavonoids. Overall, a majority of animal studies have shown differences in the comparative bioavailability of synthetic *versus* natural vitamin C, although the results varied depending on the animal model, study design and body compartments measured. In contrast, all steady state comparative bioavailability studies in humans have shown no differences between synthetic and natural vitamin C, regardless of the subject population, study design or intervention used. Some pharmacokinetic studies in humans have shown transient and small comparative differences between synthetic and natural vitamin C, although these differences are likely to have minimal physiological impact. Study design issues and future research directions are discussed.

## 1. Introduction

Vitamin C (ascorbate) is an essential water-soluble micronutrient in humans and is obtained through the diet primarily from fruits and vegetables [1]. *In vivo*, it acts as a cofactor for numerous biosynthetic enzymes required for the synthesis of amino acid-derived macromolecules, neurotransmitters and neuropeptide hormones [2], and for various hydroxylases involved in the regulation of gene transcription and epigenetics [3,4]. Vitamin C is concentrated from the plasma into the body’s organs and is found in particularly high concentrations in the pituitary and adrenal glands and in the corpus luteum [5], although skeletal muscle, brain, and liver comprise the largest body pools [6]. Most animals can synthesize vitamin C from glucose in the liver [7]; however, humans and a small selection of animal species have lost the ability to synthesize vitamin C due to mutations in the gene encoding l-gulono-γ-lactone oxidase, the terminal enzyme in the vitamin C biosynthetic pathway [8]. Therefore, an adequate and regular dietary intake is essential to prevent hypovitaminosis C and the potentially fatal deficiency disease, scurvy [9].

In the mid 1700s the Royal Navy surgeon James Lind carried out controlled dietary trials and determined that citrus fruit could cure individuals with scurvy (reviewed in [10]). However, it wasn’t until the early 1900s that experimental scurvy was first produced in guinea pigs through dietary restriction and shown to be prevented by feeding the animals fresh fruits and vegetables. In the early 1930s vitamin C was isolated from fruit and vegetables and adrenal cortex and was named “hexuronic acid”, which was shown to cure scurvy in guinea pigs and was subsequently renamed ascorbic acid to reflect its anti-scorbutic properties. Vitamin C was first chemically synthesized in 1933 [10] and, since the mid 1930s, the question of the comparative bioavailability of synthetic *versus* natural, food-derived vitamin C in animal models and human subjects has been a point of consideration.

**Figure 1:**
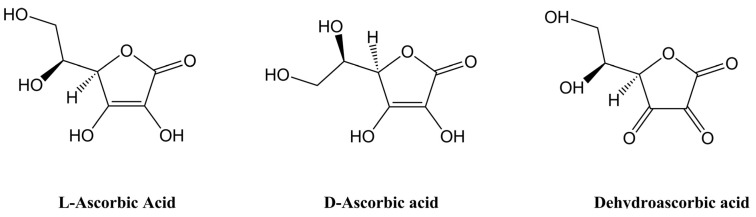
Vitamin C in its reduced form (ascorbic acid), shown as both its l- and d-isomers, and its two electron oxidation form (dehydroascorbic acid, DHA). DHA can be readily reduced back to ascorbic acid *in vivo* via both chemical and enzymatic pathways [23].

The bioavailability of dietary vitamin C represents the proportion of the micronutrient that is absorbed by the intestines and is available for metabolic processes within the body. *In vivo* vitamin C levels are a function of uptake, metabolism, and excretion (see [11] for an excellent review of these processes). Vitamin C is actively transported into the body via two sodium-dependent vitamin C transporters, SVCT1 and SVCT2 [12,13]. These transporters exhibit different tissue distributions and uptake kinetics. SVCT1 is expressed in epithelial tissues and is primarily responsible for intestinal uptake and renal reabsorption of vitamin C, the latter helping to maintain whole body homeostasis [13]. SVCT2 is expressed in specialized and metabolically active tissues and is required for delivery of vitamin C to tissues with a high demand for the vitamin either for enzymatic reactions [2] and/or to help protect these tissues from oxidative stress [13]. Both of these transporters show significantly more affinity for the l- *versus* d-isoform of vitamin C (Figure 1) [12,14], and this selectivity likely explains earlier observations of significantly lower tissue accumulation and anti-scorbutic activity of d-ascorbic acid in guinea pigs [15,16]. Although d-ascorbic acid is a commonly added food preservative [17], administration of d- and l-ascorbic acid together does not affect the bioavailability of the latter in humans [18].

Through its action as a reducing agent and antioxidant, ascorbate undergoes one and two electron oxidations to produce the ascorbyl radical and dehydroascorbic acid (DHA) (Figure 1). Recent research has shown that DHA can be taken up by the facilitative glucose transporters GLUT2 and GLUT8 in the small intestine [19]. Cells are also able to transport DHA via GLUT1 and GLUT3 [20,21], followed by intracellular reduction to ascorbate [22,23]. However, transport via the GLUTs is in competition with glucose which is at relatively high concentrations throughout the body and although different fruits and vegetables have been shown to contain relatively high amounts of DHA [24], the *in vivo* contribution of DHA is uncertain due to its minimal circulating and organ levels [25,26] (although white blood cells may be an exception to this) [27,28].

Synthetic and food-derived vitamin C is chemically identical. However, fruit and vegetables are rich in numerous micronutrients (vitamins and minerals), dietary fiber, and phytochemicals (e.g., bioflavonoids), and the presence of some of these may affect the bioavailability of vitamin C. Vitamin C has long been known to interact with vitamin E by reducing the tocopheroxyl radical and regenerating native tocopherol [29]. Some fruit, such as kiwifruit, contain relatively high amounts of vitamin E and one animal study has indicated that vitamin E is able to preserve vitamin C *in vivo* [30]. Food-derived (and synthetic) vitamin C is well known to increase non-heme iron uptake and body status, likely via its ability to reduce iron from its ferric to ferrous state [31,32]. However, whether iron can affect vitamin C bioavailability is less clear [33,34,35]. Although iron has been shown to increase the uptake of vitamin C in cultured intestinal cells [33], human intervention studies have shown no effect of iron intake on vitamin C bioavailability [34,35]. One study has indicated that specific dietary fibers, such as hemicellulose and pectin, may affect the excretion of vitamin C [36], however, their influence on vitamin C uptake was not determined.

Plant-derived flavonoids have been of interest since the mid 1930s, when they were initially referred to as “vitamin P”, primarily due to their effect on vascular permeability [37]. At the time, there was much debate in the literature regarding the role of “vitamin P” in experimental [38,39,40,41,42] and human scurvy [37,43,44,45]. Flavonoids can act as antioxidants via direct scavenging of free radicals [46,47] and/or chelation of redox-active metal ions [48,49]. As a result, it has been suggested that flavonoids may “spare” vitamin C and, thus, increase its bioavailability. Flavonoids have been shown to inhibit the *in vitro* oxidation of vitamin C [48,49,50,51], however, the *in vivo* relevance of metal-ion mediated oxidation of vitamin C is likely to be minimal as free metal ions are largely sequestered in the body [52]. Whether flavonoids can affect vitamin C uptake *in vivo* is uncertain due to the low plasma bioavailability of these compounds [53]. Thus, any interaction of flavonoids with vitamin C would be expected to occur primarily in the intestinal lumen prior to active uptake.

Of note, several *in vitro* studies have shown that various flavonoids can inhibit vitamin C and DHA uptake by their respective transporters. The flavonoid quercetin can reversibly inhibit SVCT1 expressed in *Xenopus* oocytes [54] and limited data from an animal model indicates that this may occur *in vivo* [54]. Quercetin and myricetin can inhibit the uptake of vitamin C and DHA into cultured monocytic (HL-60 and U937) and lymphocytic (Jurkat) cells via inhibition of GLUT1 and GLUT3 [55] and possibly also SVCT2, which is expressed in leukocytes [56]. Quercetin and phloretin can also inhibit the intestinal GLUT2 and GLUT8 transporters [19]. Thus, based on the above *in vitro* studies, it is unclear whether flavonoids will enhance *in vivo* vitamin C bioavailability through a sparing action, or decrease its bioavailability through inhibiting vitamin C transporters.

The effect of various purified flavonoids or flavonoid-rich fruits and vegetables on vitamin C bioavailability in different animal models and human subjects is discussed below. To test comparative vitamin C bioavailability, both steady-state and pharmacokinetic models have been used. The former monitors ascorbate levels in blood and/or urine following a number of weeks of supplementation, while the latter monitors transient changes in plasma levels and/or urinary excretion over the hours following ingestion of the vitamin C-containing test substance. The gold standard for analysis of vitamin C is HPLC with coulometric electrochemical detection due to its sensitivity and specificity [57]. Early studies, however, were limited primarily to colourimetric methods based on reduction of ferric iron compounds and are prone to interference by numerous other substances [57].

## 2. Vitamin C Bioavailability Studies Using Animal Models

There are a number of benefits to the use of animal models to investigate vitamin C bioavailability, particularly the ease of diet control and the ability to obtain tissues not normally accessible in human studies. However, results can vary widely depending on the animal model used and the different treatment and analytical methodologies employed. It should also be noted that not all of the animal models that have been used are naturally vitamin C deficient. The animal models of choice are the naturally vitamin C deficient guinea pig, and genetically scorbutic animal models, such as the Osteogenic Disorder Shionogi (ODS) rat [58], the l-gulono-γ-lactone oxidase (*Gulo*^−/−^) knockout mouse [59], and the spontaneous bone fracture (*sfx*) mouse [60]. Although animal studies can provide useful information, translation of the findings to humans should always proceed with caution.

Studies investigating the comparative bioavailability of synthetic *versus* natural vitamin C in animal models are shown in Table 1. Studies carried out in guinea pigs showed enhanced uptake of vitamin C into specific organs (e.g., adrenals and spleen) in the presence of flavonoid-rich juices/extracts or purified plant flavonoids (e.g., hesperidin, rutin, and catechin) [42,61,62,63,64]. Vinson and Bose [65] carried out a pharmacokinetic study in guinea pigs and found a 148% increase in the area under the plasma ascorbate concentration-time curve when administered as citrus fruit media. They also noted that the citrus fruit group demonstrated delayed plasma vitamin C uptake compared with the synthetic vitamin C group [65]. Cotereau *et al.* [42] reported that animals given both vitmain C and catechin not only had four to eight-fold more vitamin C in the organs measured, but they were also the only group without scorbutic-type lesions. The latter finding was supported by a similar study showing fewer fresh hemorrhages in scorbutic guinea pigs receiving vitamin C with rutin or querceitin compared with vitamin C alone [66].

Several of the studies in Table 1, however, showed no differences in vitamin C accumulation in some organs (e.g., liver) [61,62,63,67]. Hughes *et al.* noted that the acerola cherry preparation they used was virtually flavonoid free due to dilution of the high vitamin C fruit extract, which they suggested may have accounted for its reduced efficacy compared with blackcurrant juice, which is flavonoid rich [64]. To account for the flavonoid-dependent differences in vitamin C uptake observed between the adrenals and livers of guinea pigs [62,63], Douglass and Kamp [62] noted that flavonols such as rutin are rapidly destroyed in liver tissue, but are relatively stable in adrenal homogenates. Papageorge *et al.* [63] also noted that when epinephrine oxidizes it can contribute to the destruction of vitamin C and thus the antioxidant effects of rutin may result in “sparing” of vitamin C in adrenals. A study by Levine’s group [54] showed that the flavonoid quercetin can reversibly inhibit vitamin C intestinal transport and decrease plasma levels of the vitamin in the CD (Sprague-Dawley) rat, although it should be noted that this is not a vitamin C deficient animal model. Some of the variability observed in these different animal studies (Table 1) may be due to the varying ratios of flavonoid to vitamin C employed.

We recently carried out a comparative bioavailability study, using the *Gulo* mouse model, investigating the uptake of vitamin C from kiwifruit gel compared with synthetic vitamin C [68]. We found that the kiwifruit extract, which is rich in flavonoids [69,70], provided significantly higher serum, leukocyte, heart, liver, and kidney levels of vitamin C than the purified vitamin, suggesting some type of synergistic activity of the whole fruit in this model. As with Wilson *et al.* [61], we did not observe any difference between the two interventions with respect to vitamin C uptake into the brain. Indeed, there is significant retention of vitamin C in the brain during dietary depletion [64,68], suggesting a vital role for vitamin C in the brain. Thus, a significant proportion of animal studies show enhanced circulating and organ levels of vitamin C in the presence of food-derived or purified flavonoids.

## 3. Steady State Bioavailability Studies in Humans

An early report of several patients with scurvy whose plasma vitamin C levels did not increase with synthetic vitamin C alone, but only in the form of lemon juice [45], initially leant support to the “vitamin P”/flavonoid theory. However, in contrast to the animal studies, all steady state human studies (summarized in Table 2) have shown little difference in plasma and/or urine bioavailability between synthetic vitamin C and that from different fruits, fruit juices, and vegetables [35,71,72,73,74,75,76]. Mangels *et al.* [35] did observe a 20% lower plasma bioavailability of vitamin C from raw broccoli compared with cooked broccoli, however, this may have been due to differences in mechanical homogenization (chewing), a similar effect to that observed for carotenoid absorption from raw *versus* cooked carrots.

We recently carried out a steady state bioavailability study in young non-smoking men supplemented for six weeks with 50 mg/day vitamin C, in the form of a chewable vitamin C tablet or half a gold kiwifruit [77]. This dose was chosen as it lies on the steep part of the sigmoidal plasma uptake curve [78], thus enhancing the likelihood of detecting a difference between the two interventions. Although most steady state studies have used sequential or crossover study designs, we chose a randomized parallel arms design for a number of reasons. Block *et al.* [79] have previously observed a lower plasma vitamin C response to supplemental vitamin C in the second phase of a multiple depletion/repletion study. Furthermore, although washout of vitamin C could be monitored between the two phases of a cross-over study, it would not be possible to monitor washout of other kiwifruit-derived components, e.g., vitamin E, which may affect the second phase of a cross-over study due to potential *in vivo* interactions with the supplemental vitamin C [30].

Only one previous study has investigated the comparative bioavailability of synthetic *versus* natural vitamin C in leukocytes [71]. These investigators found no difference in leukocyte vitamin C uptake between synthetic vitamin C (in the presence or absence of rutin) and that in orange juice two hours after a single 75 mg dose [71]. Therefore, in addition to plasma, urine, and semen samples, we also isolated peripheral blood mononuclear cells and neutrophils before and after intervention. Due to ease of accessibility and isolation, peripheral blood leukocytes are often used as a marker for tissue vitamin C status, however, whether they are a good model for all organs and tissues is uncertain. In support of this premise our animal study indicated that different organs exhibited maximal vitamin C uptake at varying doses of the vitamin [68] and we have recently shown that human skeletal muscle exhibits greater relative uptake of vitamin C than leukocytes [80]. Therefore, we also carried out needle biopsies of skeletal muscle tissue (*vastus lateralis*), before and after intervention. In contrast to our earlier animal study [68], our human study clearly showed no differences in the steady-state bioavailability of kiwifruit-derived *versus* synthetic vitamin C to plasma, semen, peripheral blood leukocytes, and skeletal muscle tissue [77]. Thus, other nutrients and phytochemicals present in kiwifruit are neither enhancing nor inhibiting the uptake of vitamin C from the whole fruit in humans.

## 4. Pharmacokinetic Bioavailability Studies in Humans

Pharmacokinetic studies show transient changes in plasma vitamin C levels and urinary excretion over the hours following ingestion of the vitamin C-containing test substance (relevant studies are shown in Table 3). Supplemental vitamin C typically takes about two hours to reach maximal plasma levels following ingestion. An early animal study found that vitamin C provided in citrus fruit media took longer to reach peak plasma concentrations compared with a synthetic vitamin C solution and also provided a larger area under the plasma vitamin C concentration-time curve [65]. These same investigators observed a comparable trend in human subjects supplemented with 500 mg vitamin C in the presence or absence of a citrus fruit extract [81]. The citrus fruit extract delayed maximal plasma levels by one hour and provided a 35% increase in vitamin C bioavailability. Interestingly, the citrus fruit extract increased 24 h urinary vitamin C excretion in participants pre-saturated with vitamin C, but decreased excretion in non-saturated participants compared with synthetic vitamin C alone. This suggests that the baseline vitamin C status of the individual may affect the comparative bioavailability of vitamin C. Although two other studies showed increased urinary excretion in vitamin C pre-saturated subjects in the presence of fruit juice [71,82], another pre-saturation study showed comparable plasma levels and 24 h urinary excretion in the presence of mixed bioflavonoids [83]. It should be noted that doses of 500 mg vitamin C have reduced intestinal bioavailability [78] and are significantly higher than would be obtained through a normal daily diet.

A number of pharmacokinetic studies have shown comparable bioavailability of vitamin C supplied in synthetic form or in the presence of foods or fruit juices [84,85,86,87,88]. Nelson *et al.* [88] used an intestinal triple lumen tube perfusion model to investigate the absorption of synthetic vitamin C and that from an orange juice solution. This method allows direct measurement of intraluminal events and showed no difference in the absorption of vitamin C from the two test solutions. A few pharmacokinetic studies have shown transient decreases in plasma vitamin C levels and/or urinary excretion at specific time points in the presence of food and fruit juices [34,71,84,85]. The physiological relevance of these transient differences is, however, likely minimal.

We recently carried out a pharmacokinetic bioavailability study of synthetic *versus* kiwifruit-derived vitamin C in nine non-smoking males (aged 18–35 years) who had “healthy” or “optimal” (*i.e.*, >50 μmol/L) baseline levels of plasma vitamin C [89]. The participants received either a chewable tablet (200 mg vitamin C) or the equivalent dose from gold kiwifruit. Fasting blood and urine were collected half hourly to hourly over the eight hours following intervention. Plasma ascorbate levels increased from 0.5 h post intervention, although no significant differences in the plasma time-concentration curves were observed between the two interventions. An estimate of the total increase in plasma ascorbate indicated complete uptake of the ingested vitamin C tablet and kiwifruit-derived vitamin C. There was an increase in urinary ascorbate excretion, relative to urinary creatinine, from two hours post intervention. There was also a significant difference between the two interventions, with enhanced ascorbate excretion observed in the kiwifruit group. Urinary excretion was calculated as ~40% and ~50% of the ingested dose from the vitamin C tablet and kiwifruit arms, respectively. Overall, our pharmacokinetic study showed comparable relative bioavailability of kiwifruit-derived vitamin C and synthetic vitamin C [89].

**Table 1:** Vitamin C comparative bioavailability studies in animal models.

Animal Model	Intervention	Study Design	Vitamin C Analysis	Bioavailability Findings: Natural *vs.* Synthetic Vitamin C	Bioavailability Summary: Natural *vs.* Synthetic Vitamin C	Reference
Gulo^−/−^ mice	0.5–5 mg/day vitamin C solution	4 weeks intervention	HPLC-ECD	Kiwifruit ↑ serum, leukocyte, heart, liver, and kidney, but not brain vitamin C	Enhanced uptake in 5/6 pools	[68]
	Kiwifruit gel					
CD rats	60 mg/kg vitamin C gavage	Single dose; 4 h sampling	HPLC-ECD	Quercetin ↓ plasma vitamin C (at 4 h)	Decreased uptake in 1/1 pool	[54]
	15 mg/kg quercetin					
Guinea pigs	50 mg vitamin C solution	Single dose; 4 h sampling	Fluorometric (NQSA)	Citrus fruit media ↑ plasma AUC	Enhanced uptake in 1/1 pool	[65]
	Citrus fruit media					
	1 mg/kg vitamin C (low vitamin C diet)	26 days intervention	Colorimetric (DCPIP)	Orange peel extract ↑ adrenal, spleen and leukocyte, but not brain vitamin C; hesperidin ↑ adrenal and leukocyte, but not spleen vitamin C	Enhanced uptake in 3/4 pools	[61]
	50 mg/kg orange peel extract					
	50 mg/kg hesperidin					
	5 mg/kg vitamin C solution	23 days intervention	Colorimetric (DCPIP)	Black current juice ↑ adrenal and spleen vitamin C; acerola cherry juice comparable adrenal and spleen vitamin C	Enhanced uptake in 2/2 organs	[64]
	Black current juice					
	Acerola cherry juice					
	0, 5 and 10 mg/kg vitamin C	3 weeks intervention	Colorimetric (DNPH)	Rutin ↑ adrenal, but not liver vitamin C	Enhanced uptake in 1/2 pools	[62]
	50 mg rutin					
	4 mg/kg vitamin C (low vitamin C diet)	22 days intervention	Colorimetric (DNPH)	Rutin ↑ adrenal, but not liver or whole blood vitamin C of adequate animals	Enhanced uptake in 1/3 pools	[63]
	10 mg rutin tablet					
	18 mg/kg vitamin C (adequate vitamin C diet)					
	10 mg rutin tablet					
	Basic diet	23 days intervention	Colorimetric (DCPIP)	Vitamin C + catechin ↑ liver, spleen, kidney, and adrenal vitamin C	Enhanced uptake in 4/4 organs	[42]
	1 mg/animal catechin					
	10 mg/animal vitamin C					
	Vitamin C + catechin					
	0.5 mg/day vitamin C solution	20 days intervention	Colorimetric (DCPIP)	Lemon juice comparable plasma and adrenal vitamin C	Comparable uptake in 2/2 pools	[67]
	1 mL lemon juice					

NQSA: 1,2-naphthoquinone-4-sulfonic acid; AUC: area under the concentration-time curve; DNPH: 2,4-dinitrophenylhydrazine; DCPIP: 2,6-dichlorophenolindophenol.

**Table 2:** Steady state comparative bioavailability studies in humans.

Subjects	Intervention	Study Design	Vitamin C Analysis	Bioavailability Findings: Natural *vs.* Synthetic Vitamin C	Bioavailability Summary: Natural *vs.* Synthetic Vitamin C	Reference
36 non-smoking males 18–35 years	50 mg/day vitamin C tablet	6 weeks of supplementation; Parallel design	HPLC-ECD	Kiwifruit comparable plasma, urine, semen, mononuclear cell, neutrophil and muscle tissue vitamin C	Comparable uptake in 6/6 pools	[77]
	Gold kiwifruit (50 mg vitamin C)					
11 non-smoking women 21–39 years	69 mg/day vitamin C capsule	2 weeks of supplementation; Crossover design (2 week washout)	Colorimetric (DNPH)	Orange juice comparable plasma vitamin C	Comparable uptake in 1/1 pool	[76]
	Orange juice (66 mg vitamin C)					
68 non-smoking males 30–59 years	108 mg/day vitamin C tablet	4 weeks of supplementation; Crossover design (4 week washout)	Colorimetric (DNPH)	Orange pieces/juice or cooked broccoli comparable plasma vitamin C; raw broccoli ↓ plasma vitamin C	Comparable uptake in 1/1 pool	[35]
	Orange—pieces or juice					
	Broccoli—cooked or raw					
14 men and women	75 mg/day vitamin C	Sequential design	Colorimetric	Papayas and guava juice comparable plasma and urinary vitamin C	Comparable uptake in 2/2 pools	[72]
	Papayas (75 mg/day vitamin C)					
	Guava juice (75 mg/day vitamin C)					
4 healthy young subjects	75 mg/day vitamin C tablets	Pre-study saturation; Sequential design	Colorimetric	Raw cabbage and tomato juice comparable plasma and urinary vitamin C	Comparable uptake in 2/2 pools	[73]
	Raw cabbage (75 mg/day vitamin C)					
	Tomato juice (75 mg/day vitamin C)					
7 college women	40 mg/day vitamin C solution	Pre-study saturation; Sequential design	Colorimetric (DCPIP)	Raspberries comparable blood and urinary vitamin C	Comparable uptake in 2/2 pools	[74]
	Red raspberries (40 mg/day vitamin C)					
12 young adults	100 mg/day vitamin C	Sequential design	Colorimetric (DCPIP)	Orange juice comparable urinary vitamin C	Comparable uptake in 1/1 pool	[75]
	Orange juice (100 mg/day vitamin C)					

DNPH: 2,4-dinitrophenylhydrazine; DCPIP: 2,6-dichlorophenolindophenol.

**Table 3:** Pharmacokinetic comparative bioavailability studies in humans.

Subjects	Intervention	Study Design	Vitamin C Analysis	Plasma Uptake	Urinary Excretion	Reference
9 non-smoking males 18–35 years	200 mg vitamin C tablet	8 h sampling; Crossover design (3 week washout)	HPLC-ECD	Kiwifruit comparable plasma vitamin C and AUC	Kiwifruit ↑ urinary vitamin C and AUC (relative to creatinine)	[89]
	Gold kiwifruit (200 mg vitamin C)					
5 non-smoking males 22–27 years	50 mg vitamin C solution;	8 h sampling; Crossover design (4 week washout)	HPLC-ECD	Mashed potatoes ↓ plasma vitamin C (at 1 to 2.5 h); potato chips ↓ AUC	Mashed potatoes ↓ urinary vitamin C (at 3 h)	[84]
	282 g mashed potato (50 mg vitamin C)					
	87 g potato chips (50 mg vitamin C)					
	Placebo					
5 non-smoking males 22–26 years	50–500 mg vitamin C solution	6 h sampling; Crossover design	HPLC-ECD	Acerola juice comparable plasma vitamin C and AUC	Acerola juice ↓ urinary vitamin C (at 1, 2 and 5 h)	[85]
	100 mL acerola juice (50 mg vitamin C)					
12 males 20–35 years	284 mg vitamin C drink	4.5 h sampling; Crossover design (1 week washout)	Colorimetric (TPTZ)	Orange juice comparable bioavailability (AUC/concentration)	ND	[86]
	590 mL orange juice (68 mg vitamin C)					
	Placebo (milk)					
7 non-smoking females	150 mg vitamin C solution	8 h sampling; Crossover design (2 week washout)	HPLC-UV	Orange juice comparable plasma vitamin C	ND	[87]
	300 mL orange juice (150 mg vitamin C)					
	Placebo					
7 non-smokers 26–59 years	30 mg vitamin C solution	4 h sampling; Crossover design (3–4 week washout)	Fluorometric (phenylene diamine)	Grape juice ↓ plasma vitamin C (at 16 to 28 min)	ND	[34]
	200 mL red grape juice (30 mg vitamin C)					
9 healthy subjects 19–41 years	500 mg vitamin C tablet	1 g/day vitamin C for 2 weeks pre-study; 8 h sampling; Crossover design (1 week washout)	Colorimetric (DNPH)	Bioflavonoids comparable AUC	Bioflavonoids comparable 24 h vitamin C excretion	[83]
	Mixed bioflavonoids					
	Placebo					
12 non-smoking subjects 18–41 years	500 mg vitamin C solution	Subgroup had 1 g/day vitamin C for 2 weeks pre-study; 8 h sampling; Crossover design (1 week washout)	Fluorometric	Citrus extract ↑ AUC	Citrus extract ↓ 24 h vitamin C excretion in non-saturated subjects and ↑ 24 h vitamin C excretion in saturated subjects	[81]
	2 g citrus extract					
	Placebo					
5 men 21–25 years	500 mg vitamin C solution	100 mg/day vitamin C for 1 month pre-study; 8 h sampling; Crossover design (1 week washout)	Colorimetric (Indophenol dye)	ND	Blackcurrant juice slight ↑ 8 h vitamin C excretion in saturated subjects	[82]
	500 mg vitamin C in blackcurrant juice					
15 normal subjects (4 smokers) 20–42 years	70 mg/h vitamin C solution	Intestinal perfusion; Tandem design	Colorimetric (DNPH)	Orange juice comparable intestinal absorption	ND	[88]
	Orange juice					
12 men (6 smokers) 23–44 years	75 mg vitamin C solution	Pre- and post-saturation with 1 mg/day vitamin C; 2–24 h sampling; Crossover design (1 day washout)	Colorimetric	Orange juice and rutin ↓ plasma vitamin C (at 2 h)	Orange juice slight ↑ 24 h vitamin C excretion	[71]
	400 mg rutin					
	Orange juice (75 mg vitamin C)					

AUC: area under the concentration-time curve; ND: not determined; DNPH: 2,4-dinitrophenylhydrazine; TPTZ: 2,4,6-tris(2-pyridyl)-*s*-triazine.

## 5. Vitamin C Bioavailability from Different Tablet Formulations

Doses of vitamin C up to 2000 mg/day are considered safe for general consumption [90]. However, pharmacokinetic studies indicate that ingestion of single doses of vitamin C greater than 200 mg have lower relative bioavailability [78], indicating that ingestion of several smaller doses each day is preferable to a single large dose. A number of studies have investigated the relative bioavailability of vitamin C from different tablet formulations and have shown that slow-release formulations provide superior vitamin bioavailability [91,92,93,94]. Salts of vitamin C, such as sodium and calcium ascorbate (Ester-C), have also been tested. Animal studies indicated that Ester-C (which contains calcium ascorbate, as well as DHA and calcium threonate) was absorbed more readily and excreted less rapidly than ascorbic acid [95] and had superior anti-scorbutic activity in ODS rats [96]. Johnston and Luo [83], however, found no significant differences between Ester-C and ascorbic acid bioavailability in humans. Nevertheless, Ester-C has been shown to be better tolerated in individuals sensitive to acidic foods [97].

## 6. Conclusions

Overall, a majority of animal studies have shown differences in the comparative bioavailability of synthetic *versus* food-derived vitamin C, or vitamin C in the presence of isolated bioflavonoids, although the results varied depending on the animal model, study design and body compartments measured. In contrast, all steady state comparative bioavailability studies in humans have shown no differences between synthetic and natural vitamin C, regardless of the subject population, study design or intervention used. Some pharmacokinetic studies in humans have shown transient and small comparative differences between synthetic and natural vitamin C, although these differences are likely to have minimal physiological impact. Thus, not only do the reviewed studies reiterate the injunction that the findings of animal studies should not be directly translated to humans [98,99], but it is also apparent that additional comparative bioavailability studies in humans are unwarranted.

Although synthetic and food-derived vitamin C appear to be equally bioavailable in humans, ingesting vitamin C as part of a whole food is considered preferable because of the concomitant consumption of numerous other macro- and micronutrients and phytochemicals, which will confer additional health benefits. Numerous epidemiological studies have indicated that higher intakes of fruit and vegetables are associated with decreased incidence of stroke [100], coronary heart disease [101], and cancers at various sites [102,103]. Vitamin C status is one of the best markers for fruit and vegetable intake [104], and food-derived vitamin C is associated with decreased incidence of numerous chronic diseases [1], however, whether the observed health effects of fruit and vegetable ingestion are due to vitamin C and/or other plant-derived components is currently unknown. With respect to coronary heart disease, strong evidence exists for a protective effect of vegetables, moderate evidence for fruit and dietary vitamin C and insufficient evidence for supplemental vitamin C [105]. Some meta-analyses support the premise that dietary vitamin C is more protective than supplements [106], while others show reduced disease incidence with supplemental but not dietary vitamin C [107].

A major limitation with epidemiological studies is that they show only an association between dietary vitamin C intake and disease risk and cannot ascertain whether different sources of vitamin C (*i.e.*, food-derived *versus* supplement) are a cause, consequence, or simply a correlate of the particular end-point measured. Interpretations can also vary significantly depending on the input of different confounders [108]. Furthermore, epidemiological studies rely predominantly on food frequency questionnaires [109,110] and 24 h dietary recalls [111] to ascertain vitamin C intakes from foods and/or supplements [112]. This methodology has numerous limitations [113] and correlations with vitamin C status can vary depending on the methods employed as well as numerous other external factors [114]. Pooled or meta-analyses of epidemiological studies are particularly problematic due to the combining of variable study designs, cohorts and endpoints, often resulting in dilution or misinterpretation of study findings.

The gold standard for determining causality is the double-blind randomized placebo controlled clinical trial. Although this type of study design works well for comparing the effects of drugs against a placebo, it does not work for nutrients, such as vitamin C, which are already in the food chain and are required for life, *i.e.*, there is no true placebo. Numerous other methodological issues have been identified with the design of many clinical trials investigating the health effects of vitamin C [115]. For example, a major flaw with many vitamin C intervention studies is the use of study populations with already adequate or even saturating vitamin C levels, which significantly decreases the likelihood of observing any effects of the intervention. Thus, it is recommended that study populations are comprised of individuals with sub-optimal vitamin C status (*i.e.*, <50 μmol/L plasma vitamin C) or that sub-group analysis is carried out on the low vitamin C sub-populations [116]. With pharmacokinetic studies, both unsaturated and saturated individuals can be used, but comparative bioavailability studies have shown that results may vary depending on the baseline vitamin C status of the study subjects. Furthermore, the vitamin C doses chosen for intervention are critical since doses above 200 mg have decreased intestinal uptake [78], indicating that if higher doses are warranted then these should be provided as multiple doses of ~200 mg each to ensure complete bioavailability.

The comparative health effects of supplemental *versus* food-derived vitamin C will only be determined through the use of appropriate and well-designed studies. Determination of the physiological effects or health outcomes of intervention with synthetic *versus* natural vitamin C will depend largely on the endpoints measured. Only a handful of comparative intervention studies have been carried out to assess specific physiological or health endpoints. Guarnieri *et al.* [89] investigated potential protection of mononuclear leukocytes from supplemented individuals against *ex vivo* oxidative DNA damage. Although they found comparable vitamin C bioavailability between a single portion of orange juice (containing 150 mg vitamin C) and a synthetic vitamin C drink of the same dosage, they showed that only the orange juice protected the leukocytes from *ex vivo* oxidative DNA damage [89]. However, how closely *ex vivo* oxidation of DNA resembles events occurring *in vivo* is debatable and results could also vary significantly depending on the type of oxidative stress. Johnston *et al.* [76] compared plasma lipid peroxidation in individuals who had been supplemented with either orange juice or synthetic vitamin C (~70 mg/day) for two weeks. They found comparable vitamin C bioavailability and a similar reduction in lipid peroxidation with both interventions [76]. Several studies have assessed the effects of synthetic and natural vitamin C, or vitamin C in the presence of bioflavonoids, on the common cold. Two earlier studies showed a lack of an effect of vitamin C (~200 mg/day), with and without purified bioflavonoids, on the prevention and cure of the common cold [117,118]. Another study indicated that synthetic vitamin C (80 mg/day) and orange juice both decreased the symptoms of the common cold compared with placebo, but there were no differences between the two interventions [119].

As alluded to in the introduction, vitamin C is known to enhance the bioavailability of other nutrients, such as vitamin E [30] and non-heme iron [31,32], which may enhance the health effects of vitamin C-containing foods. Bioflavonoids are also known to have numerous biological activities [120]. Recently vitamin C has been shown to modulate specific biological activities of quercetin and tea polyphenols [121,122]. Thus, future studies may elucidate the physiological relevance of these interactions.
